# Classification of new morbillivirus and jeilongvirus sequences from bats sampled in Brazil and Malaysia

**DOI:** 10.1007/s00705-022-05500-z

**Published:** 2022-07-04

**Authors:** Heather L. Wells, Elizabeth Loh, Alessandra Nava, Mónica Romero Solorio, Mei Ho Lee, Jimmy Lee, Jum R. A. Sukor, Isamara Navarrete-Macias, Eliza Liang, Cadhla Firth, Jonathan H. Epstein, Melinda K. Rostal, Carlos Zambrana-Torrelio, Kris Murray, Peter Daszak, Tracey Goldstein, Jonna A. K. Mazet, Benhur Lee, Tom Hughes, Edison Durigon, Simon J. Anthony

**Affiliations:** 1 Department of Ecology, Evolution, and Environmental Biology, Columbia University, New York, NY USA; 2 EcoHealth Alliance, New York, NY USA; 3 Division of Natural Sciences and Mathematics, Transylvania University, Lexington, KY USA; 4 Instituto Leônidas and Maria Deane, Fiocruz Amazônia, Manaus, Brazil; 5 Department of Preventive Veterinary Medicine and Animal Health, School of Veterinary Medicine, University of São Paulo, São Paulo, Brazil; 6 Conservation Medicine, Sungai Buloh, Selangor Malaysia; 7 Sabah Wildlife Department, Kota Kinabalu, Sabah Malaysia; 8 Department of Pathology, Microbiology, and Immunology, School of Veterinary Medicine, University of California Davis, Davis, CA USA; 9 Center for Infection and Immunity, Mailman School of Public Health, Columbia University, New York, NY USA; 10 Department of Environmental Science and Policy, George Mason University, Fairfax, VA USA; 11 School of Public Health, Imperial College London, London, UK; 12 MRC Unit The Gambia at London, School of Hygiene and Tropical Medicine, Atlantic Boulevard, Fajara, The Gambia; 13 Zoological Pathology Program, University of Illinois at Urbana-Champaign, Brookfield, IL USA; 14 One Health Institute and Karen C. Drayer Wildlife Health Center, School of Veterinary Medicine, University of California, Davis, CA USA; 15 Icahn School of Medicine at Mount Sinai, New York, NY USA; 16 Institute of Biomedical Science, University of São Paulo, São Paulo, Brazil

## Abstract

As part of a broad One Health surveillance effort to detect novel viruses in wildlife and people, we report several paramyxovirus sequences sampled primarily from bats during 2013 and 2014 in Brazil and Malaysia, including seven from which we recovered full-length genomes. Of these, six represent the first full-length paramyxovirid genomes sequenced from the Americas, including two that are the first full-length bat morbillivirus genome sequences published to date. Our findings add to the vast number of viral sequences in public repositories, which have been increasing considerably in recent years due to the rising accessibility of metagenomics. Taxonomic classification of these sequences in the absence of phenotypic data has been a significant challenge, particularly in the subfamily *Orthoparamyxovirinae*, where the rate of discovery of novel sequences has been substantial. Using pairwise amino acid sequence classification (PAASC), we propose that five of these sequences belong to members of the genus *Jeilongvirus* and two belong to members of the genus *Morbillivirus*. We also highlight inconsistencies in the classification of Tupaia virus and Mòjiāng virus using the same demarcation criteria and suggest reclassification of these viruses into new genera. Importantly, this study underscores the critical importance of sequence length in PAASC analysis as well as the importance of biological characteristics such as genome organization in the taxonomic classification of viral sequences.

**Supplementary Information:**

The online version contains supplementary material available at 10.1007/s00705-022-05500-z.

## Introduction

**Fig. 1:**
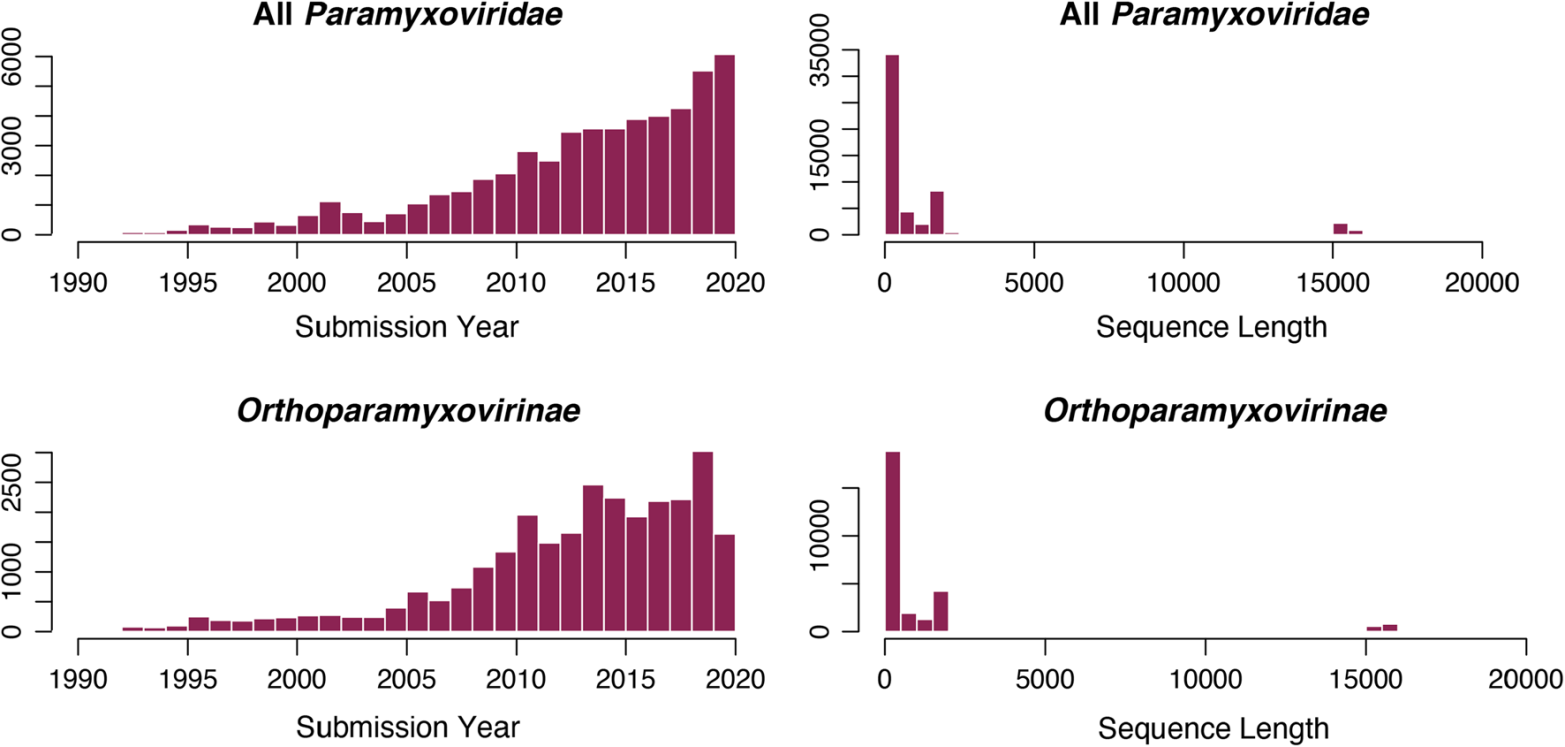
Histograms of paramyxovirid sequences submitted to GenBank by submission year (left) and sequence length (right). Trends are shown for all *Paramyxoviridae* (top) and *Orthoparamyxovirinae* only (bottom). The number of sequences submitted is increasing each year, but a significant majority of sequences submitted are of short PCR fragments (~500 bp), and very few are of full genomes (~15-16 kb).

With the recent emergence of zoonotic paramyxovirids, including Nipah virus and Hendra virus, a great deal of effort has been placed on discovering novel paramyxovirids in wildlife [1–3]. However, increased surveillance over the last decade has revealed a multitude of novel bat- and rodent-borne paramyxovirids that do not fall into previously defined genera. This issue has been particularly problematic within the subfamily *Orthoparamyxovirinae*, where most newly discovered sequences are phylogenetically positioned between the well-established genera *Morbillivirus* and *Henipavirus* [4]. Recently, two new genera have been created to encompass a number of these sequences: *Jeilongvirus* and *Narmovirus* [5] (Fig. 1).

Classically, paramyxovirid taxonomy has relied on phenotypic differences such as the presence of neuraminidase and/or hemagglutination activity to demarcate distinct taxonomic groups [4, 6, 7]. However, the rate of detection of new viruses through genetic sequencing has now vastly outpaced the rate at which these viruses can be isolated and experimentally characterized, necessitating a new taxonomic approach. New frameworks for virus classification emphasize a wider biological context, such as host range, genome organization and presence of additional transcriptional units (ATUs), pairwise amino acid sequence comparison (PAASC), and cell receptor usage [4, 6]. Recently, several authors have suggested the creation of a new paramyxovirid genus, “*Shaanvirus*”, to encompass a group of sequences that are related to jeilongviruses but have a distinct genome organization and host range [8–10]. These new viruses have been sampled from bats (with the exception of Belerina virus, which was found in a hedgehog) as opposed to rodents, which were previously the only host group known to harbor jeilongviruses [11–14], and have non-homologous ORFs between the fusion protein and receptor binding protein (RBP) genes in the genome. This classification was later rejected by the ICTV in favor of placing these viruses in the existing genus *Jeilongvirus* due to sequence relatedness in the RNA-dependent RNA polymerase (RdRp) gene, despite the differences in other characteristics [15].

Here, we report the sequences of seven new paramyxovirid genomes from bats: two from members of the genus *Morbillivirus* from Brazil, one closely related to members of the proposed genus “*Shaanvirus*” from Sabah on Malaysian Borneo, and four additional sequences from Brazil that form a monophyletic group and have biological features that are distinct from those of members of any of the established genera. We found that a modified version of the pairwise amino acid sequence comparison (PAASC) tool does not support the classification of these four viruses from Brazil as members of a new genus, despite the distinct characteristics of this clade. Instead, our investigations support the classification of these four novel viruses as members of the genus *Jeilongvirus*, along with viruses previously proposed to constitute the genus “*Shaanvirus*”*.* As a result, the genus *Jeilongvirus* would consist of several monophyletic subclades with distinct characteristics. We propose labeling each of these clades as a way of distinguishing between groups of viruses that are closely related but with differing genome organizations, geographic origins, and host ranges. While distinct, these groups still have the defining commonality of additional transcriptional units in the genome that are not present in other orthoparamyxovirins, which is thus far unique to the genus *Jeilongvirus*. We also highlight a number of considerations for the existing classification of other orthoparamyxovirins, primarily that the classification of Mòjiāng virus and Tupaia virus is inconsistent with proposed genus demarcation cutoffs, and these species should therefore be placed into new genera. Critically, we also show that previous limitations to using PAASC as a tool to classify sequences can be resolved when specific regard is given to the length of the sequences being tested.

## Materials and methods

### Field sampling and laboratory screening for paramyxovirids

Rectal, oral, blood, and urine samples from bats, rodents, or non-human primates in Brazil and Sabah, Malaysia were collected previously as part of a larger research effort [16] and analyzed for this study (Supplementary Tables S1 and S2). Extraction of total nucleic acid and conversion to cDNA were performed as described previously [17, 18]. Consensus PCR (cPCR) assays using paramyxovirid-specific degenerate primers were used to screen for samples positive for paramyxovirids as described by Tong et al. [19]. The degenerate primers bind two highly conserved regions with variable sequence between them, allowing for broad reactivity within the viral family and detection of both known and novel viruses. PCR products were screened by gel electrophoresis, and bands corresponding to the expected amplicon size were cloned using a StrataClone PCR cloning kit and sequenced by the Sanger method to confirm the presence of paramyxovirids. Partial gene sequences from positive samples were deposited in the GenBank database (Supplementary Tables S1 and S2).

### Genome sequencing of paramyxovirid-positive samples

Total nucleic acid of 39 paramyxovirid-positive samples was sequenced using an Illumina HiSeq platform. Quality control and adapter trimming were performed on the resulting reads using Cutadapt v1.18, and host reads were subtracted using Bowtie v2.3. 5 (*Phyllostomus*: NCBI Genome ID 75334; *Carollia* and *Diaemus*: 22833, *Hipposideros*: 75235; *Myotis*: 43810). The resulting reads were assembled *de novo* using MEGAHIT v1.2.8 [20]. Contigs were scaffolded to a reference sequence (morbilliviruses: GenBank accession AF014953; jeilongviruses: KC154054) and any overlaps or gaps were confirmed by iterative local alignment using Bowtie2 [21]. The full genome sequences were deposited in the GenBank database (Table 1), and sequencing files are available on SRA (Supplementary Table S3).

**Table 1:** Full-length viral genome sequences described in this study and their suggested taxonomic classification, host species, geographic origin (plus latitude/longitude, where available), collection date, and associated GenBank accession numbers

Virus	Classification	Host species	Geographic origin	Collection date	GenBank accession no.
PDF-3137	*Morbillivirus*	*Myotis riparius*	Brazil (Amazon) -2.39, -60.05	12/03/2013	MW557651 MW554523 (host COI) MW557650 (host CytB)
PBZ-1381	*Morbillivirus*	*Phyllostomus hastatus*	Brazil (Atlantic Forest) -22.25, -52.18	06/10/2013	MZ312422 MZ312423 (host CytB)
PDF-0699	*Jeilongvirus –* “Subclade B”	*Hipposideros galeritus*	Malaysia 5.53, 118.08	12/13/2012	MZ312421
PDF-3308	*Jeilongvirus –* “Subclade D”	*Carollia perspicillata*	Brazil (Amazon) -2.41, -59.41	01/03/2014	MZ312420
PBZ-1672	*Jeilongvirus –* “Subclade D”	*Carollia perspicillata*	Brazil (Atlantic Forest) -22.57, -52.30	03/05/2013	MZ312428 MZ312429 (host CytB)
PBZ-3205	*Jeilongvirus –* “Subclade D”	*Diaemus youngi*	Brazil (Atlantic Forest) -22.25, -52.18	10/01/2013	MZ312424 MZ312425 (host CytB)
PBZ-2282	*Jeilongvirus –* “Subclade D”	*Diaemus youngi*	Brazil (Atlantic Forest) -22.49, -52.39	09/12/2013	MZ312426 MZ312427 (host CytB)

### Phylogenetic analysis

To reconstruct the phylogenetic history of the seven novel genomes, we first collected from GenBank all orthoparamyxovirin sequences for which the full genome was available as well as those for which the majority of the RdRp (previously polymerase [L]) gene was available. We excluded members of the genus *Respirovirus*, as none of our sequences fell within this group, but we included Sendai virus as an outgroup to root the phylogeny. Phylogenetic reconstruction was first performed using all available full- or nearly full-length RdRp nucleotide sequences, which were aligned relative to their amino acid translations using MUSCLE (32 sequences and 8,048 bp, see Supplementary material). Maximum-likelihood trees were generated using IQTREE (v1.6.12) with built-in model estimation and 100 bootstrap replicates [22]. Phylogenetic trees were also built for each of the other genes individually (N, P, M, F, and RBP) using the same methods to compare topologies for any inconsistencies. Six sequences for which only the polymerase gene was available were excluded from these trees.

### Pairwise amino acid sequence comparison (PAASC)

All sequences of orthoparamyxovirins classified to the species level for which any length of RdRp sequence was available in the GenBank database were used for PAASC analysis. These data consisted of several long sequences, but the majority of sequences in GenBank are very short fragments (~500 bp) generated by common PCR assays used for virus discovery [19] (Fig. 1, right). Because these fragments are from assays targeting different regions and do not overlap, an alignment was created manually. First, all full- or nearly full-length RdRp sequences were aligned. Second, Tong-PanPMV (a PCR assay targeting all paramyxovirids) and Tong-RMH (a PCR assay targeting sequences from members of the genera *Respirovirus*, *Morbillivirus*, and *Henipavirus*) were each aligned separately. Finally, an alignment with one full-length RdRp sequence and one sequence of either Tong-PanPMV or Tong-RMH was used as a reference with which to position the fragment region alignments joined onto the full RdRp alignment backbone. Because there are no sequence gaps in the fragment regions of the alignments, this method is robust due to its manual nature. The alignment is available as Supplementary Material.

Pairwise distances between sequences were determined using nucleotide percent identity or BLOSUM62 matrix scores and used to build histograms demonstrating the distributions of pairwise identities (PIDs) between viruses classified within the same species, in different species within the same genus, or in species within two different genera. This approach is in contrast to the NCBI-PASC tool, which uses a BLAST-based comparison calculation [23]. This analysis was performed for nucleotide sequences as well as amino acid sequences. Where fragments did not overlap, no pairwise comparisons were made. For the short-fragment histograms, only the corresponding regions were used within sequences for which a full- or nearly full-length RdRp sequence was available. For amino acid sequence alignments, percentages were calculated as the BLOSUM62 alignment score divided by the total possible score (i.e., 100% identity).

## Results

cPCR screening of samples collected in Brazil identified 15 novel paramyxovirid sequences from bats of six different species (*Carollia perspicillata, Diaemus youngi, Myotis riparius, Phyllostomus elongatus, P. hastatus*, and *Pteronotus parnellii*) (Supplementary Table S1). Based on their RdRp gene phylogeny, two sequences, one from *P. hastatus* and one from *M. riparius*, clustered with members of the genus *Morbillivirus* (Supplementary Fig. S1). The remaining sequences clustered within an unnamed sister clade to the lineage including members of the genus *Jeilongvirus* and the proposed genus “*Shaanvirus*”, with the exception of one sequence from *P. parnellii*. This sequence clustered within the same group but was positioned on a single long branch (Supplementary Fig. S1). This phylogeny also demonstrated that viruses included in the proposed genus “*Shaanvirus*” were paraphyletic and formed two separate clades, here referred to as “shaanvirus clade 1” and “shaanvirus clade 2” for clarity (but not for official taxonomic naming purposes).

In Sabah, Malaysia, cPCR screening identified an additional 24 novel paramyxovirid sequences from bats of six additional species (*Hipposideros cervinus, H. diadema, H. galeritus, Rhinolophus arcuatus, R. creaghi,* and *R. trifoliatus*) and one sequence from a moonrat (*Echinosorex gymnura*), an animal that is not closely related to rodents and belongs to the hedgehog family (Supplementary Table S2). All of these sequences clustered within either “shaanvirus clade 1” or “shaanvirus clade 2” in the RdRp gene phylogeny (Supplementary Fig. S1). The single sequence from *E. gymnura* is most closely related to Belerina virus, which was isolated from a European hedgehog (*Erinaceus europaeus*).

**Fig. 2:**
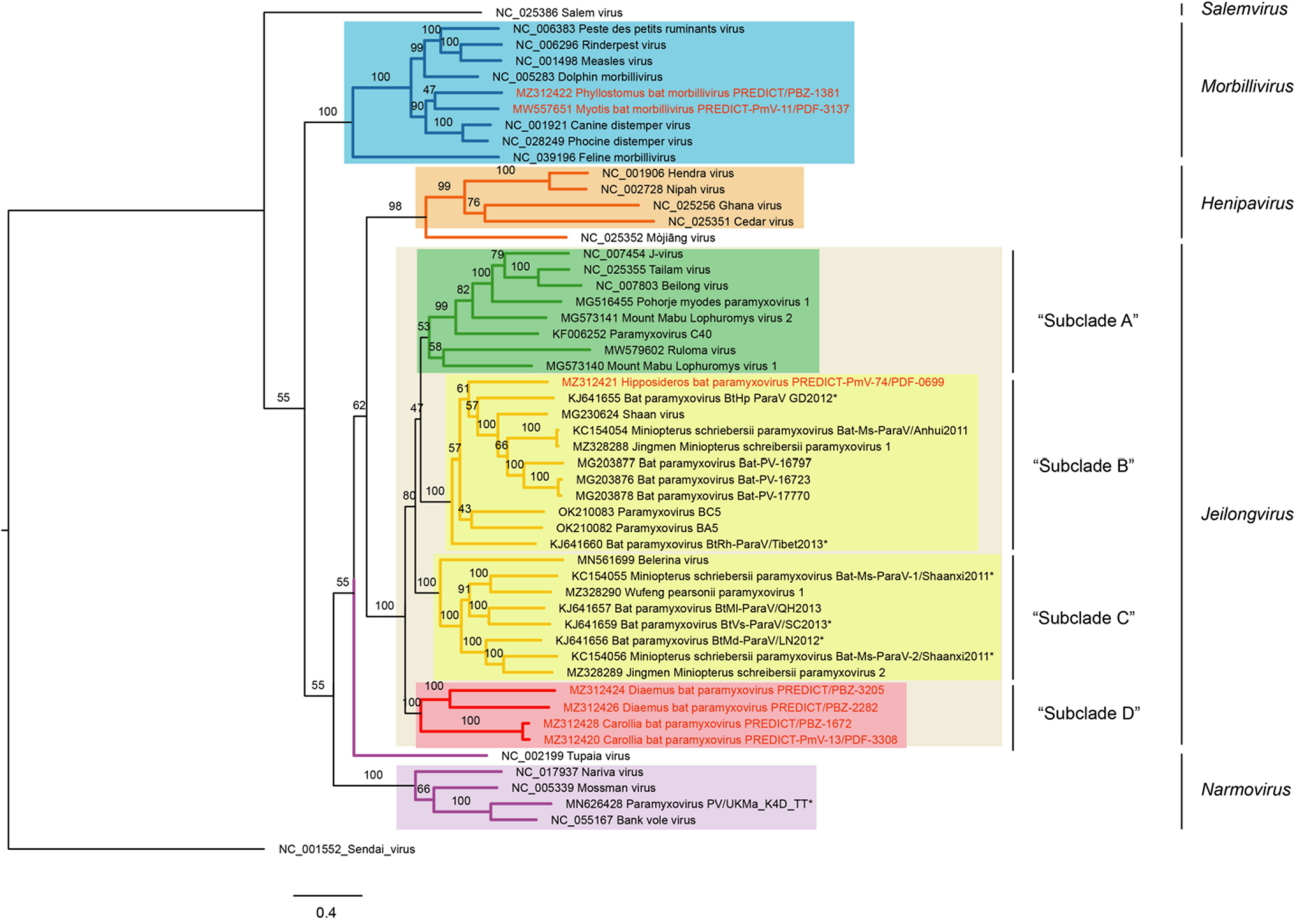
Nucleotide-sequence-based phylogeny with maximum-likelihood bootstrap values of the orthoparamyxovirins for which full- or nearly full-length RdRp gene sequences are available. The genus *Respirovirus* is represented by a single sequence, that of Sendai virus, as an outgroup. Branches are colored according to their current genus classifications, which are also indicated at the right. Colored highlighting indicates the taxonomic suggestions discussed here, including reclassifying Tupaia virus and Mòjiāng virus each into its own new genus and establishing the subclades A-D within the genus *Jeilongvirus*.

**Fig. 3:**
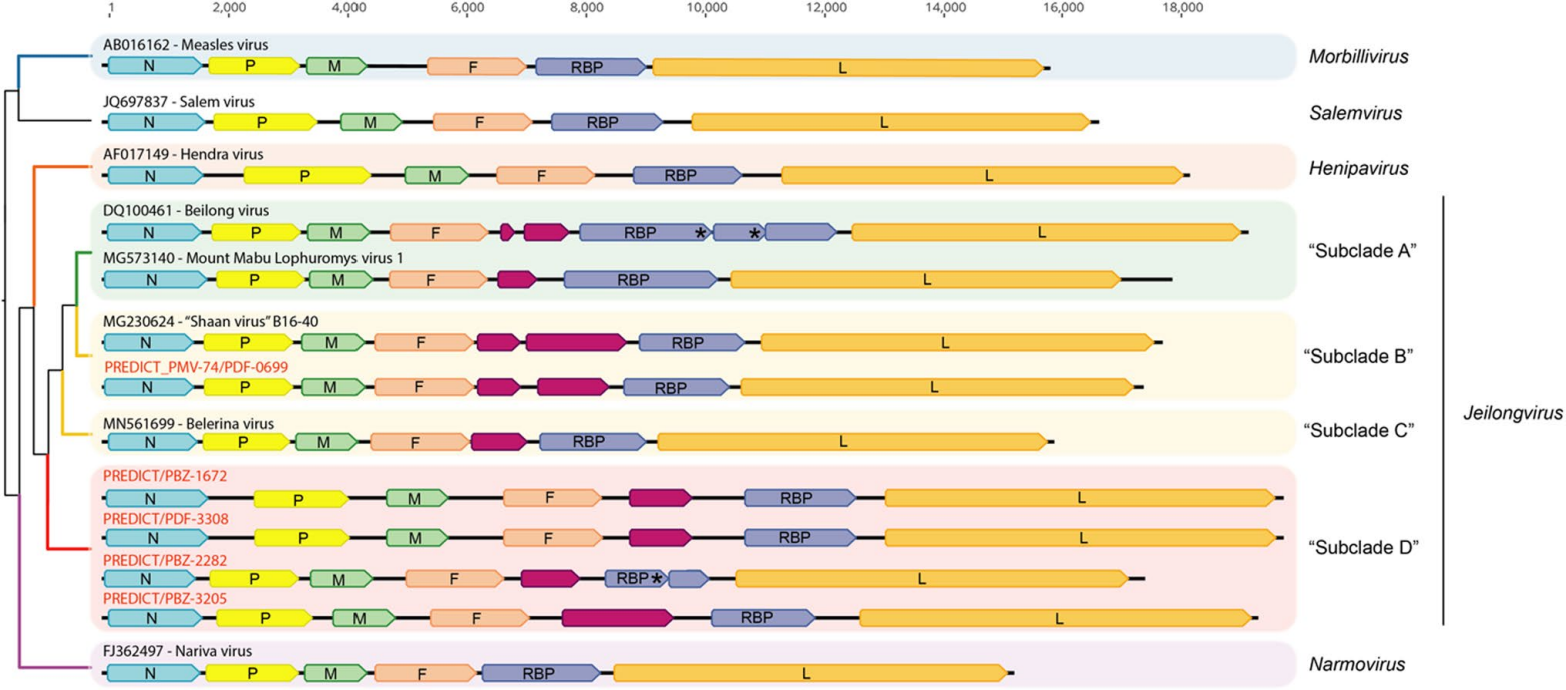
Genome organization of representative members of each genus included in this study. Sequences are organized according to their RdRp phylogeny, which is shown on the left. The new genome sequences described here are labeled with red font, with the exception of the bat morbilliviruses (PDF-3137 and PBZ-1381), which share the same genome arrangement with other morbilliviruses. Where more than one genome arrangement is present in a single genus, a representative of each type is shown. ORFs are represented by colored polygons, and the black lines represent the entire length of the genome, both of which are drawn to scale. ORFs are colored as follows: nucleocapsid (N gene), blue; phosphoprotein (P gene), yellow; matrix (M gene), green; fusion (F gene), peach; ATUs, magenta; receptor binding protein (RBP gene), purple; RNA-dependent RNA polymerase (RdRp gene), orange. Asterisks on RBP ORFs indicate premature stop codons.

In total, 39 cPCR-positive samples were analyzed by high-throughput sequencing. From these, we recovered seven novel genome sequences (Table 1). PDF-3137 and PBZ-1381 from Brazil belong to members of the genus *Morbillivirus*, clustering most closely with canine distemper virus and phocine distemper virus in the RdRp gene phylogeny (Fig. 2). PDF-3137 was found in a sample from a *Myotis riparius* bat (family *Molossidae*) in the Amazon rainforest, while PBZ-1381 was found in a sample from a *Phyllostomus hastatus* bat (family Phyllostomidae) in the Atlantic Forest. Both have the same genome arrangement as other morbilliviruses (Fig. 3), and the virus corresponding to PDF-3137 has also been shown to utilize the morbillivirus receptors SLAMF1/NECTIN4 [24]. The single sequence from Sabah, Malaysia, PDF-0699, was found in a sample obtained from a *Hipposideros galeritus* bat. This sequence was most closely related to sequences in “shaanvirus clade 2” and has the same two ATUs between those encoding the fusion and receptor-binding proteins (Figs. 2 and 3). The remaining four sequences from Brazil, PDF-3308, PBZ-1672, PBZ-3205, and PBZ-2282, comprise a monophyletic group (Fig. 2). These four unclassified sequences were each characterized by the presence of a single ATU in the middle of the genome (Fig. 3). A BLASTx search of the NCBI database using the ATU sequence from each genome resulted in no hits to any other published sequence in GenBank. The ATUs of PDF-3308 and PBZ-1672 appear to be homologous and share 92% sequence identity, while PBZ-3205 and PBZ-2282 share <40% sequence identity with any of the other three sequences and only 25% identity to each other, suggesting that they are not homologous. PDF-3308 and PBZ-1672 were found in *Carollia perspicillata*, while PBZ-3205 and PBZ-2282 were found in *Diaemus youngi*, both in the family Phyllostomidae.

**Fig. 4:**
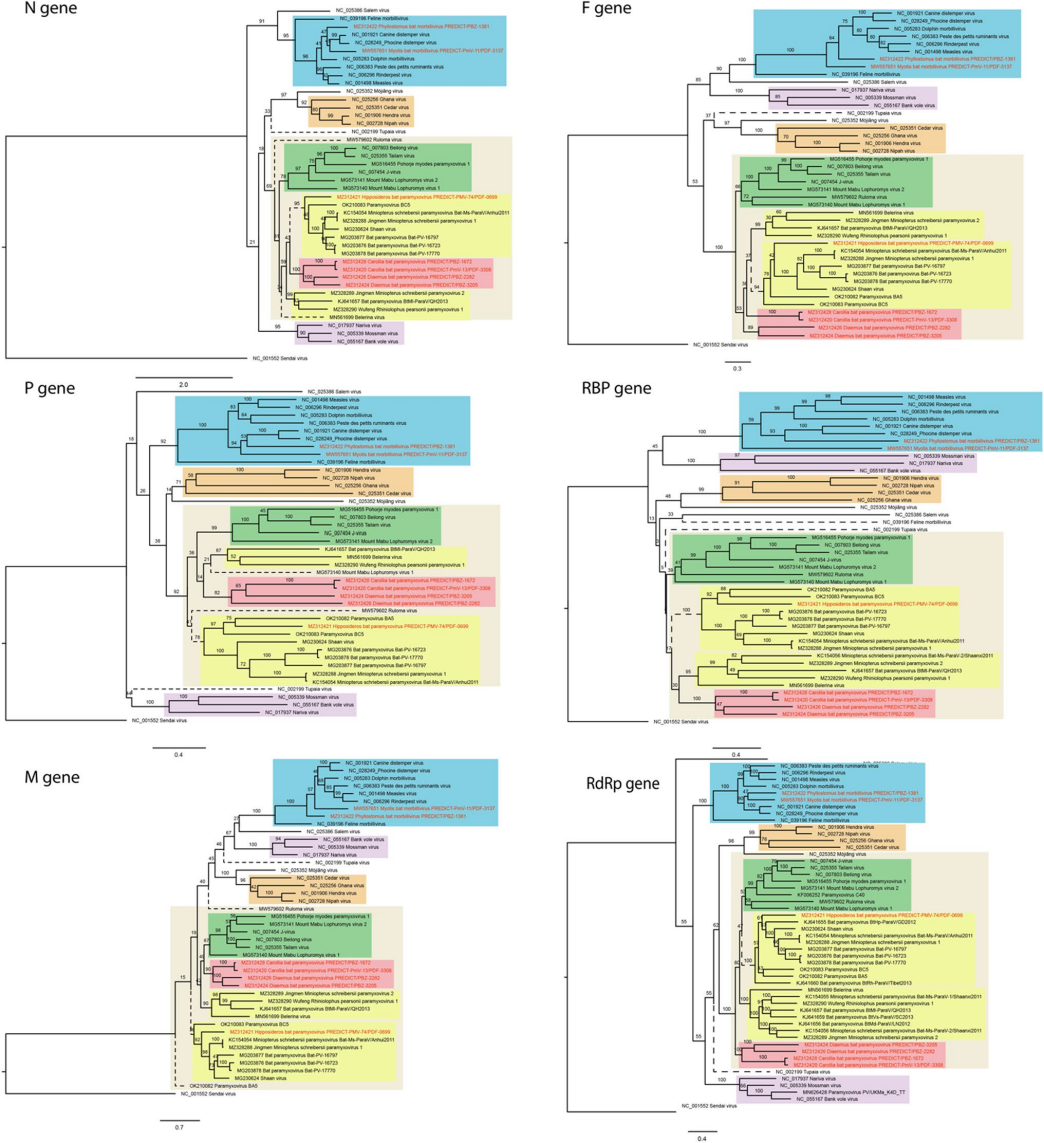
Nucleotide-sequence-based phylogenies for all genes, excluding ATUs. Clades are color-coded with the same scheme as in Fig. 2: *Morbillivirus*, blue; *Henipavirus*, orange; *Jeilongvirus* “subclade A”, green; *Jeilongvirus* “subclades B” and “C”, yellow; *Jeilongvirus* “subclade D”, red; *Narmovirus*, purple. Where sequences are incomplete, no sequence is shown in the tree for that gene. For clades that are not monophyletic by genus in one or more of the trees (i.e., for paraphyletic sequences previously proposed as “shaanviruses” and for Tupaia virus), branches are shown as dotted lines.

We also examined the topology of the phylogenies constructed from the other five genes and found that these new sequences from Brazil consistently clustered as a monophyletic clade (Fig. 4). In addition, “shaanvirus clade 1” and “shaanvirus clade 2” sequences were also paraphyletic in phylogenies constructed based on all five other genes of the genome (Fig. 4). Similarly, we found that the species *Tupaia virus* is not monophyletic with respect to the other narmovirus sequences for the N, M, F, and RBP genes, where it resides independently on a single long branch (Fig. 4).

**Fig. 5:**
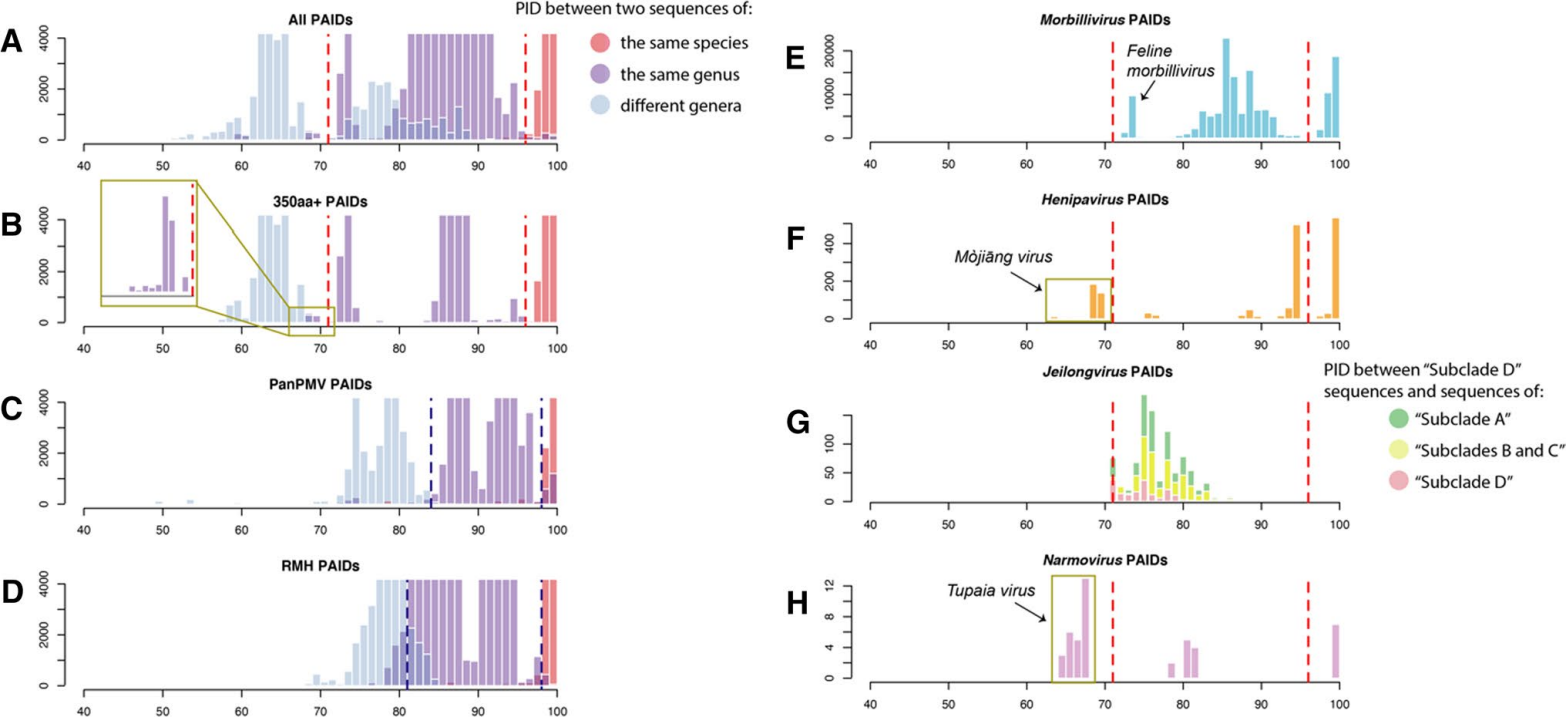
PAASC histograms of pairwise amino acid comparisons based on the RdRp gene. Scores on the *x*-axis were calculated by scoring alignments with the BLOSUM62 matrix and dividing by the total possible score (100% identity). Values on the *y*-axis indicate frequency. Gold boxes correspond to PIDs between sequences that do not conform to the identified cutoff values based on their established classifications. The cutoff values used are shown as dotted lines, with red for full-length RdRp sequences and blue for short fragments (Tong-PanPMV and Tong-RMH).

To support classification of the four genomes from Brazil, we implemented PAASC at the nucleotide and amino acid levels. As no distinct cutoffs have been published for taxon demarcation within the family *Paramyxoviridae* [4], we first performed PAASC on all existing classified orthoparamyxovirins belonging to the ICTV-approved genera *Morbillivirus*, *Henipavirus*, *Jeilongvirus*, and *Narmovirus* to compare the distributions of PIDs for sequences of members of the same species, of the same genus, and of different genera (Fig. 5). The distribution of pairwise amino acid identities (PAIDs) for sequences from members of the same viral species is well separated from the distribution of PAIDs between members of different species above and below 96%; however, there is significant overlap in distributions of PAIDs between sequences from members of the same genus and sequences from members of different genera between 70 and 90% (Fig. 5A). We suspect that this may be due to the fact that many sequences submitted to GenBank are generated using the same commonly used consensus PCR assays, Tong-PanPMV and Tong-RMH. Since these assays each target a small, conserved region of the RdRp gene, PIDs may be biased upwards when only this small fragment is available for comparison. Indeed, we found that when we looked at PAIDs only between sequences with 350 or more amino acids, the distributions became much more distinct (Fig. 5B). We also generated histograms for each of the two consensus PCR assay fragments (Tong-PanPMV and Tong-RMH), which demonstrated that the average PAID between sequences from members of different genera shifted upwards from ~65% to nearly 80%, eliminating the distinction observed when only long sequences are considered (Fig. 5C and D).

Although the histogram of PAIDs for sequences greater than 350 aa showed a significant improvement in demarcation, there is a small peak of PAIDs between sequences from members of the same genus that fall closer to the distribution of PAIDs between sequences from members of different genera (Fig. 5B; gold box). To investigate this peak, we performed the same PAASC analysis for each genus individually. The PAIDs of three of the established genera (*Morbillivirus*, *Henipavirus,* and *Narmovirus*) mainly show distributions that would be expected for sequences from members of the same species or genus, but *Henipavirus* and *Narmovirus* each contain a small cluster of sequences distinctly less similar to the rest of the distribution (Fig. 5F and H; gold boxes). We identified these PAIDs as henipavirus sequences when compared with Mòjiāng virus and narmovirus sequences when compared with Tupaia virus. A cluster of low similarity was also observed for morbillivirus sequences when compared with feline morbillivirus (Fig. 5E). We decided to place a cutoff value at 71% for two viruses to belong to the same genus, as this results in all PAIDs between sequences of members of different genera falling below the cutoff and the majority of sequences from members of the same genus falling above this cutoff. This cutoff value supports the inclusion of feline morbillivirus in the genus *Morbillivirus* but places Mòjiāng virus and Tupaia virus outside the genera *Henipavirus* and *Narmovirus*, respectively.

Sequences of members of the genus *Jeilongvirus* all have PAIDs that fall above the cutoff. “Shaanvirus” sequences in both clades 1 and 2 have PAIDs >73%, which would place the two in the same genus despite the fact that they are not monophyletic and have different genome arrangements. Further, PAIDs between “shaanvirus” sequences, jeilongvirus sequences, or the four novel sequences from Brazil compared to any other are all >71%, which would technically place each of these groups into a single genus despite vastly different biological characteristics (Fig. 5G). Thus, the viruses in the “shaanvirus” group as well as those from Brazil would be classified as belonging to the genus *Jeilongvirus* according to the cutoffs established here.

Identical analyses were performed at the nucleotide level, and the distributions of pairwise nucleotide identities (PNIDs) resulted in the same inferences as those of the PAIDs. The cutoffs for nucleotides were found to lie at approximately 55% and 80% for genus and species, respectively.

## Discussion

Pairwise amino acid sequence comparison (PAASC) analysis has previously been suggested as a tool to rapidly classify viruses when only the genetic sequence is available [23]. When pairwise identity values form distinct distributions separable by a single cutoff value, this analysis can be very effective. With paramyxovirids, however, no such cutoffs have yet been determined, and considerable difficulty has been highlighted in attempts to do so [4]. Here, we performed PAASC analysis separately from the NCBI platform and used only the conserved RdRp gene. Using all available sequences in GenBank, we found considerable overlap in distributions of PIDs between sequences from members of the same genus or of different genera, with no single cutoff value appearing appropriate for delineating between the two, which is in agreement with the results obtained using NCBI-PAASC tool. However, we found that by separating PIDs by the length of the sequences being compared, much clearer cutoff values can be attained. Because the small sequence fragments prevalent in GenBank were generated using a consensus PCR assay that targets a conserved region of the genome, the PID comparisons using these sequences are biased upwards considerably. By restricting the sequences used to determine cutoff values to those with more than 350 aa (or 1000 nt) in length, we demonstrate complete separation in sequence comparisons within the same genus and between different genera, with a considerable gap between them, significantly reducing the ambiguity in cutoff placement. When considering PIDs between sequences shorter than this length, our cutoff values were shifted upwards by 4-6% and showed a much less generous distinction between members of the same or different genera, but this may still be useful for classification where PIDs for the sequence in question are not near these cutoff values. One of the most important findings of this study is that short fragment sequences in GenBank should not be considered for analysis using the NCBI PAASC analysis tool, and that cutoff values of 71% for amino acid sequence comparisons and 55% for nucleotide sequence comparisons are appropriate at the genus level.

Relying only on genetic sequence and biological characteristics, we have provided evidence to support the classification of four novel paramyxoviruses (PDF-3308, PBZ-1672, PBZ-3205, and PBZ-2282) as members of the genus *Jeilongvirus*. This evidence also supports the decision made by the ICTV to classify viruses in the “shaanvirus” group as members of the genus *Jeilongvirus*. This classification is based on PAASC analysis, which shows that these four viruses do not meet our defined cutoff criteria to be considered members of a novel genus (71% PAID or 55% PNID) when compared to established jeilongvirus sequences. Classification within a single genus is supported by the fact that these groups comprise the only orthoparamyxovirins that have ATUs in the middle of the genome. However, the distinct characteristics of each, such as host taxa and geographic range, underline the need for further distinction. As such, we also suggest that a labeling system be adopted for individual clades within the genus *Jeilongvirus* to distinguish groups with different characteristics, including host range, geographic distribution, and genome arrangement. For example, the earliest discovered members of the genus *Jeilongvirus,* including the namesake members Beilong virus and J-virus*,* are found exclusively in rodents, while sequences from the “shaanvirus” group and our novel group from Brazil are primarily found in bats, underscoring distinct evolutionary trajectories. The geographic range in South America is also unique to the four novel sequences from Brazil reported here, where no other complete genomes of paramyxovirids from bats have been sequenced or classified to date. We suggest that the clade containing Beilong virus and J-virus be considered “subclade A”, that “shaanvirus clade 1” and “shaanvirus clade 2” be considered “subclades B” and “C”, respectively, and that the clade containing sequences from Brazil be considered “subclade D”. We also show that the sequence from Sabah, Malaysia, PDF-0699, shares PIDs consistent with its inclusion in “subclade C”, and it has the same arrangement of ATUs in the genome as other members of this clade.

An additional outcome of this study is the first description of the complete genome sequences of bat morbilliviruses (PDF-3137 and PBZ-1381), both sampled from Brazil. The finding that PDF-3137 shares the same receptor usage as all other morbilliviruses, using SLAMF1 and NECTIN4, further supports its classification as a member of the genus *Morbillivirus* [24] (referred to as MBaMV in the previous publication). The only other known bat morbillivirus sequences were also collected from phyllostomid bats in Brazil but were not completely sequenced [2].

In performing our analysis to determine PAASC cutoff values, we found that pairwise comparisons of sequences from Mòjiāng virus and Tupaia virus with those from members of the same genus resulted in values that fell below our suggested cutoff values (Fig. 5). This finding raises the question as to whether these sequences should actually be considered members of the genera to which they currently belong. PAASC is just one tool that can be used to taxonomically classify these sequences, and other characteristics should be considered to determine if a virus should be included in an existing genus, such as receptor usage, host range, and the presence of ATUs (Table 2). For example, feline morbillivirus PAIDs fall extremely close to the 71% cutoff, but this virus is very similar to other morbilliviruses in its other characteristics. Morbilliviruses have a broad host range, use SLAM1/NECTIN4 cell receptors, and have no ATUs – all of which apply to feline morbillivirus, which also clusters monophyletically with other morbilliviruses in every gene. The same does not apply to Tupaia virus and the other members of the genus *Narmovirus*. The cell receptor usage of members of this genus is currently not known, but Tupaia virus does not cluster within the *Narmovirus* clade in phylogenetic analysis based on the N, M, F, and RBP genes, and it falls below the 70% PAID cutoff in PAASC analysis. Mòjiāng virus also shows significant deviation from the traits of other henipaviruses. Mòjiāng virus is rodent-borne and is unable to use ENFB2/3 as a receptor, whereas other members of this genus are bat-borne and use ENFB2/3. Mòjiāng virus is consistently monophyletic with other henipaviruses, but PAASC analysis also places this sequence below the 70% PAID cutoff. While the characteristics of feline morbillivirus are in line with other morbilliviruses despite its lower PIDs, the same is not true for Mòjiāng virus and Tupaia virus, which should have their current genus classifications reconsidered. We propose the creation of a new genus for each of these viruses.

**Table 2:** Genera of the paramyxovirus subfamily *Orthoparamyxovirinae*, with the exception of *Salemvirus* and *Ferlavirus*, which have only one classified member, and *Respirovirus* and *Aquaparamyxovirus*, which are outside the scope of this study. For each genus, the host taxa, host cellular receptor (if known), and the number of ATUs are shown. In addition, if any classified member of that genus represents an exception to those traits, it is listed along with the differing trait(s) in the last column

Genus	Host	Receptor	ATUs	Exceptions
*Morbillivirus*	Mammals	SLAMF1/NECTIN4	None	
*Henipavirus*	Bats	ENFB2/3	None	Mòjiāng virus: host is a rodent, does not use ENFB2/3, falls below PAASC cutoff
*Jeilongvirus –* “Subclade A”	Rodents	Unknown	Two	
*Jeilongvirus –* “Subclade B”	Bats	Unknown	Two	
*Jeilongvirus –* “Subclade C”	Bats	Unknown	One	Belerina virus: host is a hedgehog
*Jeilongvirus –* “Subclade D”	Bats	Unknown	One	
*Narmovirus*	Rodents	Unknown	None	Tupaia virus: does not share monophyly, falls below PAASC cutoff

Taxonomic classification, particularly when it comes to viruses, is a task that requires continuous reevaluation and flexibility to keep up with new information when it becomes available. Previous classifications within the family *Paramyxoviridae* have changed several times in recent years with the discovery of new sequences, and the suggestions outlined here will undoubtedly come under review with the addition of new information in the future. The taxonomic changes described here are presented for discussion, but they have not been endorsed by the ICTV Executive Committee and may ultimately differ from those that are approved. The avalanche of new sequences and information emerging for paramyxovirids each year sets a pace that can quickly negate our best classification efforts. Future research should focus on determining the functions of the unknown ATUs in many of these genomes and on receptor usage of viruses for which it is still unknown, which will help illuminate borderline cases. Should researchers wish to embark on classification schemes using only PAASC, careful consideration should be given to ensuring that the cutoff values being used are appropriate for the length of the sequences being compared. This is highlighted by our finding that PID distributions generated by short or long sequence lengths are considerably inconsistent. Finally, we emphasize the critical importance of the generation of full genome sequences where possible when describing novel viruses from wildlife that were first identified using cPCR. In cases where genome sequencing is successful, the additional information that can be gleaned from the genome sequence is extremely valuable not only for classification of new sequences but also for refinement of existing taxonomic classifications.

## Supplementary Information

Below is the link to the electronic supplementary material.

Supplementary file1 (DOC 804 kb)

Supplementary file2 (FASTA 411 kb)

Supplementary file3 (FASTA 5398 kb)

## Data Availability

All sequences reported in this study have been deposited in the GenBank database, and the alignments used for analysis are included as supplementary materials.
