# Supplementary material for: Classification of new morbillivirus and jeilongvirus sequences from bats sampled in Brazil and Malaysia
Source: Arch Virol. 2022 Jul 4;167(10):1977–87. doi: 10.1007/s00705-022-05500-z (PMC9402765; doi:10.1007/s00705-022-05500-z)
Supplement: Supplementary file 1 — Supplementary file1 (DOC 804 kb) [file 705_2022_5500_MOESM1_ESM.doc]

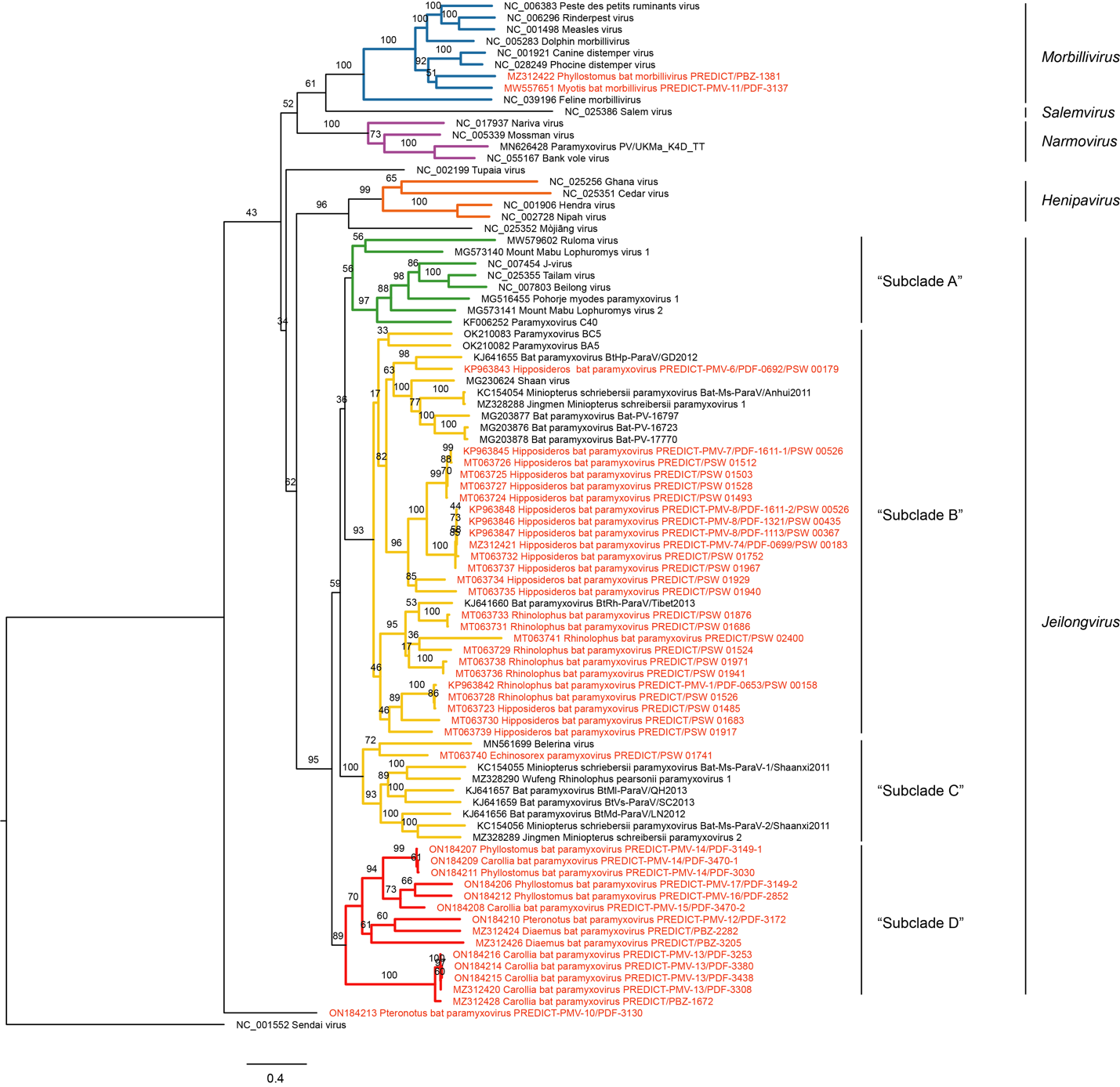


**Supplementary Fig. S1** Phylogeny of all cPCR fragments (Tong-PanPMV) sequenced as part of this study with other classified paramyxovirid sequences. Names highlighted in red are those sequenced in this study, and those indicated by an asterisk are those for which full genomes were recovered. Clade bar colors are consistent with the taxonomic classifications in Figure 1. Some maximum-likelihood bootstrap values near the tips of the tree have been removed for clarity.

**Supplementary Table S1** List of bat species sampled in Brazil, number of individuals tested from each species, and number of individuals found to be positive for paramyxovirids. Percentages in parentheses are the positivity rate for that species. Names of viruses identified and the corresponding GenBank accession numbers are also shown. Note that multiple virus names and GenBank accession numbers shown on the same line indicate coinfection of a single individual.

| Species | Individuals sampled | Individuals positive | Specimen ID | GenBank accession no. |
| --- | --- | --- | --- | --- |
| *Ametrida centurio* | 1 |  |  |  |
| *Anoura caudifer* | 1 |  |  |  |
| *Artibeus cinereus* | 1 |  |  |  |
| *Artibeus concolor* | 1 |  |  |  |
| *Artibeus fimbriatus* | 87 |  |  |  |
| *Artibeus gnomus* | 1 |  |  |  |
| *Artibeus lituratus* | 364 |  |  |  |
| *Artibeus obscurus* | 11 |  |  |  |
| *Artibeus planirostris* | 361 |  |  |  |
| *Caluromys philander* | 2 |  |  |  |
| *Carollia benkeithi* | 1 |  |  |  |
| *Carollia brevicauda* | 2 |  |  |  |
| *Carollia perspicillata* | 397 | 6 (1.5%) | PDF-3308* PDF-3438 PDF-3380 PDF-3253 PDF-3470-1, PDF-3470-2 PBZ-1672* | MZ312420  ON184215  ON184214  ON184216  ON184209, ON184208  MZ312428 |
| *Chiroderma doriae* | 3 |  |  |  |
| *Chrotopterus auritus* | 3 |  |  |  |
| *Dermanura cinereus* | 1 |  |  |  |
| *Dermanura gnoma* | 1 |  |  |  |
| *Desmodus rotundus* | 8 |  |  |  |
| *Diaemus youngi* | 5 | 2 (40%) | PBZ-3205*  PBZ-2282* | MZ312426  MZ312424 |
| *Didelphis marsupialis* | 38 |  |  |  |
| *Eptesicus brasiliensis* | 1 |  |  |  |
| *Eumops glaucinus* | 6 |  |  |  |
| *Glossophaga soricina* | 6 |  |  |  |
| *Lasiurus blosevillii* | 6 |  |  |  |
| *Lonchophylla thomasi* | 3 |  |  |  |
| *Lophostoma silvicolum* | 3 |  |  |  |
| *Marmosops sp.* | 2 |  |  |  |
| *Mesomys hispidus* | 2 |  |  |  |
| *Mesophylla macconnelli* | 2 |  |  |  |
| *Micoureus demerarae* | 25 |  |  |  |
| *Micronycteris hirsuta* | 2 |  |  |  |
| *Mimon crenulatum* | 25 |  |  |  |
| *Molossops temminckii* | 2 |  |  |  |
| *Molossus molossus* | 14 |  |  |  |
| *Monodelphis brevicaudata* | 1 |  |  |  |
| *Myotis albescens* | 13 |  |  |  |
| *Myotis riparius* | 46 | 1 (2.1%) | PDF-3137* | MW557651 |
| *Noctilio albiventris* | 1 |  |  |  |
| *Oecomys sp.* | 3 |  |  |  |
| *Philander opossum* | 20 |  |  |  |
| *Phylloderma stenops* | 2 |  |  |  |
| *Phyllostomus discolor* | 8 |  |  |  |
| *Phyllostomus elongatus* | 7 | 2 (28.6%) | PDF-3130 PDF-3172 | ON184213  ON184210 |
| *Phyllostomus hastatus* | 18 | 1 (5.6%) | PBZ-1381* | MZ312422 |
| *Platyrrhinus lineatus* | 10 |  |  |  |
| *Platyrrhinus recifinus* | 14 |  |  |  |
| *Platyrrhinus sp.* | 1 |  |  |  |
| *Proechimys sp.* | 5 |  |  |  |
| *Pteronotus parnellii* | 52 | 3 (5.8%) | PDF-3030 PDF-2852 PDF-3149-1, PDF-3149-2 | ON184211  ON184212  ON184207, ON184206 |
| *Pygoderma bilabiatum* | 8 |  |  |  |
| *Rhinophylla fischerae* | 8 |  |  |  |
| *Rhinophylla pumilio* | 23 |  |  |  |
| *Saccopteryx bilineata* | 1 |  |  |  |
| *Stunira lilium* | 190 |  |  |  |
| *Sturnira lilium* | 6 |  |  |  |
| *Sturnira tildae* | 7 |  |  |  |
| *Tonatia saurophila* | 3 |  |  |  |
| *Trachops cirrhosus* | 2 |  |  |  |
| *Trinycteris nicefori* | 1 |  |  |  |
| *Uroderma bilobatum* | 3 |  |  |  |
| *Vampyrum spectrum* | 1 |  |  |  |

**Supplementary Table S2** List of bat species sampled in Sabah, Malaysia, number of individuals tested from each species, and number of individuals found to be positive for paramyxovirids. Percentages in parentheses are the positivity rate for that species. Names of viruses identified and corresponding GenBank accession numbers are also shown. Note that multiple virus names and GenBank accession numbers shown on the same line indicate coinfection of a single individual. Viruses indicated by an asterisk are those for which the full genome sequence was determined.

| Species | Individuals sampled | Individuals positive | Specimen ID | GenBank accession no. |
| --- | --- | --- | --- | --- |
| *Arctogalidia trivirgata* | 1 |  |  |  |
| *Balionycteris maculata* | 30 |  |  |  |
| *Callosciurus adamsi* | 2 |  |  |  |
| *Callosciurus notatus* | 72 |  |  |  |
| *Callosciurus prevostii* | 3 |  |  |  |
| *Chaerephon plicatus* | 21 |  |  |  |
| *Cheiromeles torquatus* | 1 |  |  |  |
| *Chironax melanocephalus* | 5 |  |  |  |
| *Chiropodomys gliroides* | 3 |  |  |  |
| *Cynopterus brachyotis* | 67 |  |  |  |
| *Cynopterus horsfieldii* | 25 |  |  |  |
| *Cynopterus sphinx* | 4 |  |  |  |
| *Dremomys everetti* | 1 |  |  |  |
| *Dyacopterus spadiceus* | 5 |  |  |  |
| *Echinosorex gymnura* | 12 | 1 (8.3%) | PSW 01741 | MT063740 |
| *Elephas maximus borneensis* | 4 |  |  |  |
| *Glischropus tylopus* | 6 |  |  |  |
| *Helarctos malayanus* | 46 |  |  |  |
| *Hipposideros cervinus* | 50 | 10 (20%) | PSW 01485 PSW 01493 PSW 01503 PSW 01512 PSW 01528 PSW 01683 PSW 01752 PSW 01929 PSW 01940 PSW 01967 | MT063723 MT063724 MT063725 MT063726 MT063727 MT063730 MT063732 MT063734 MT063735 MT063737 |
| *Hipposideros cineraceus* | 21 |  |  |  |
| *Hipposideros diadema* | 132 | 2 (1.5%) | PDF-0692/PSW 00179 PSW 01917 | KP963843 MT063739 |
| *Hipposideros dyacorum* | 3 |  |  |  |
| *Hipposideros galeritus* | 394 | 4 (1.0%) | PDF-0699/PSW 00183* PDF-1113/PSW 00367  PDF-1321/PSW 00435 PDF-1611-1/PSW 00526, PDF-1611-2/PSW 00526 | MZ312421 KP963847 KP963846 KP963845, KP963848 |
| *Hipposideros ridleyi* | 1 |  |  |  |
| *Hylobates funereus* | 15 |  |  |  |
| *Hylobates muelleri* | 1 |  |  |  |
| *Hystrix crassispinis* | 4 |  |  |  |
| *Kerivoula intermedia* | 9 |  |  |  |
| *Kerivoula lenis* | 3 |  |  |  |
| *Kerivoula hardwickii* | 6 |  |  |  |
| *Kerivoula minuta* | 2 |  |  |  |
| *Kerivoula papillosa* | 8 |  |  |  |
| *Kerivoula pellucida* | 10 |  |  |  |
| *Lariscus hosei* | 1 |  |  |  |
| *Leopoldamys sabanus* | 47 |  |  |  |
| *Macaca nemestrina* | 22 |  |  |  |
| *Macroglossus minimus* | 40 |  |  |  |
| *Manis javanica* | 159 |  |  |  |
| *Maxomys alticola* | 3 |  |  |  |
| *Maxomys surifer* | 36 |  |  |  |
| *Maxomys whiteheadi* | 81 |  |  |  |
| *Megaderma lyra* | 1 |  |  |  |
| *Megaderma spasma* | 5 |  |  |  |
| *Megaerops ecaudatus* | 1 |  |  |  |
| *Miniopterus australis* | 6 |  |  |  |
| *Miniopterus paululus* | 4 |  |  |  |
| *Miniopterus schriebersii* | 1 |  |  |  |
| *Mops mops* | 3 |  |  |  |
| *Murina suilla* | 10 |  |  |  |
| *Murina cyclotis* | 2 |  |  |  |
| *Mydaus javanensis* | 1 |  |  |  |
| *Myotis horsfieldii* | 1 |  |  |  |
| *Nasalis larvatus* | 30 |  |  |  |
| *Niviventer cremoriventer* | 83 |  |  |  |
| *Nycteris tragata* | 1 |  |  |  |
| *Nycticebus menagensis* | 1 |  |  |  |
| *Paguma larvata* | 3 |  |  |  |
| *Paradoxurus hermaphroditus* | 8 |  |  |  |
| *Penthetor lucasi* | 20 |  |  |  |
| *Petaurillus hosei* | 2 |  |  |  |
| *Phoniscus atrox* | 1 |  |  |  |
| *Phoniscus jagorii* | 1 |  |  |  |
| *Pipistrellus coromandra* | 4 |  |  |  |
| *Pongo pygmaeus* | 58 |  |  |  |
| *Presbytis rubicunda* | 3 |  |  |  |
| *Pycnonotus plumosus* | 1 |  |  |  |
| *Rattus argentiventer* | 2 |  |  |  |
| *Rattus baluensis* | 1 |  |  |  |
| *Rattus exulans* | 4 |  |  |  |
| *Rattus norvegicus* | 1 |  |  |  |
| *Rattus rattus* | 9 |  |  |  |
| *Rattus tanezumi* | 12 |  |  |  |
| *Rattus tiomanicus* | 5 |  |  |  |
| *Rhinolophus acuminatus* | 2 |  |  |  |
| *Rhinolophus affinis* | 1 |  |  |  |
| *Rhinolophus arcuatus* | 3 | 1 (33.3%) | PSW 01971 | MT063738 |
| *Rhinolophus creaghi* | 556 | 6 (1.1%) | PDF-0653/PSW 00158 PSW 01524 PSW 01526 PSW 01686 PSW 01876 PSW 01941 | KP963842  MT063729 MT063728 MT063731 MT063733 MT063736 |
| *Rhinolophus luctus* | 1 |  |  |  |
| *Rhinolophus malayanus* | 1 |  |  |  |
| *Rhinolophus paradoxolophus* | 10 |  |  |  |
| *Rhinolophus phillippinensis* | 37 |  |  |  |
| *Rhinolophus pusillus* | 1 |  |  |  |
| *Rhinolophus sedulus* | 14 |  |  |  |
| *Rhinolophus stheno* | 1 |  |  |  |
| *Rhinolophus trifoliatus* | 54 | 1 (1.9%) | PSW 02400 | MT063741 |
| *Rhinosciurus laticadatus* | 1 |  |  |  |
| *Rousettus amplexicaudatus* | 6 |  |  |  |
| *Sundamys infraluteus* | 10 |  |  |  |
| *Sundamys muelleri* | 137 |  |  |  |
| *Sundasciurus brookei* | 1 |  |  |  |
| *Sundasciurus hippurus* | 10 |  |  |  |
| *Sundasciurus lowii* | 28 |  |  |  |
| *Tarsius bancanus* | 3 |  |  |  |
| *Tragulus javanicus* | 1 |  |  |  |
| *Tragulus napu* | 1 |  |  |  |
| *Trichys fasciculata* | 2 |  |  |  |
| *Tupaia belangeri* | 2 |  |  |  |
| *Tupaia longipes* | 13 |  |  |  |
| *Tupaia glis* | 72 |  |  |  |
| *Tupaia mino* | 1 |  |  |  |
| *Tupaia tana* | 36 |  |  |  |
| *Viverra tangalunga* | 22 |  |  |  |

**Supplementary Table S3** Accession numbers in the NCBI Sequence Read Archive (SRA) of fastq files obtained from sequenced samples

| Virus | Accession |
| --- | --- |
| PDF-3137 | SRX14843684 |
| PBZ-1381 | SRX14843687 |
| PDF-0699 | SRX14843686 |
| PDF-3308 | SRX14843685 |
| PBZ-1672 | SRX14843690 |
| PBZ-3205 | SRX14843688 |
| PBZ-2282 | SRX14843689 |

**Supplementary Material 1** FASTA file containing aligned RdRp nucleotide sequences used to generate the phylogeny in Figure 2.

**Supplementary Material 2** FASTA file containing aligned RdRp amino acid sequences used to generate the pairwise identity histograms in Figure 5.

**Supplementary Material 3** FASTA file containing aligned cPCR (Tong-PanPMV) RdRp nucleotide sequences used to generate the phylogeny in Supplementary Figure S1.
